# Al-Mg-MoS_2_ Reinforced Metal Matrix Composites: Machinability Characteristics

**DOI:** 10.3390/ma15134548

**Published:** 2022-06-28

**Authors:** Rajesh Shanmugavel, Narmada Chinthakndi, Mayakannan Selvam, Naganandhan Madasamy, Senthil Kumar Shanmugakani, Anish Nair, Chander Prakash, Dharam Buddhi, Saurav Dixit

**Affiliations:** 1 Department of Mechanical Engineering, Kalasalingam Academy of Research and Education, Krishnankoil 626126, India; s.rajesh@klu.ac.in (R.S.); narmada.ch315@gmail.com (N.C.); anishn@live.com (A.N.); 2 Department of Automobile Engineering, Kalasalingam Academy of Research and Education, Krishnankoil 626126, India; mayakannan205@gmail.com (M.S.); naganandhan1126476@gmail.com (N.M.); 3 Department of Mechanical Engineering, SRM Institute of Science and Technology, Tiruchirappalli Campus, Tiruchirappalli 621105, India; senthilkr.d19@gmail.com; 4 School of Mechanical Engineering, Lovely Professional University, Phagwara 144411, India; 5 Division of Research and Development, Lovely Professional University, Phagwara 144411, India; 6 Division of Research & Innovation, Uttaranchal University, Dehradun 248007, India; dbuddhi@gmail.com; 7 Peter the Great St. Petersburg Polytechnic University, 195251 Saint Petersburg, Russia

**Keywords:** Al-Mg-MoS_2_ composites, WEDM, EDAS, surface roughness, overcut, pulse on time

## Abstract

Several components are made from Al-Mg-based composites. MoS_2_ is used to increase the composite’s machinability. Different weight percent (3, 4, and 5) of MoS_2_ are added as reinforcement to explore the machinability properties of Al-Mg-reinforced composites. The wire cut electrical discharge machining (WEDM) process is used to study the machinability characteristics of the fabricated Al-Mg-MoS_2_ composite. The machined surface’s roughness and overcut under different process conditions are discussed. The evaluation-based distance from average solution (EDAS) method is used to identify the optimal setting to get the desired surface roughness and overcut. The following WEDM process parameters are taken to determine the impact of peak current, pulse on time, and gap voltage on surface roughness, and overcut. The WEDM tests were carried out on three different reinforced samples to determine the impact of reinforcement on surface roughness and overcut. The surface roughness and overcut increase as the reinforcement level increases, but the optimal parameters for all three composites are the same. According to EDAS analysis, I_3_, Ton_2_, and V_1_ are the best conditions. Furthermore, peak current and pulse on-time significantly influence surface roughness and overcut.

## 1. Introduction

Composite materials have existed since the dawn of humanity, but only after World War II did they became commercially feasible. A composite material comprises several distinct physical combinations to obtain new materials and enhanced properties compared to the base matrix materials [1]. The property of the fabricated composite material depends upon the fabrication route. Samal et al. [2] concluded that their powder metallurgy is suitable for manufacturing aluminum metal matrix composites (AMMC). AMMC is becoming a popular material for aircraft, automobiles, and other engineering applications. AMMCs continuously satisfy the demand for durable, lightweight, and high-performance components. AMMCs possess superior strength, wear resistance, low thermal expansion, and electrical conductivity. It helps to replace conventional aluminum alloys.

There has been a significant surge in AMMC research in the previous two to three decades. Due to their outstanding characteristics and availability, Al and Mg are commonly employed in the fabrication of metal matrix composites (MMCs). When Al-Si-Mg composites are reinforced with zircon and alumina particles, Kumar and Venkatesh [3] observe that the corrosion resistance of the composites is greatly improved. Pai et al. [4] found that in wrought alloys (6XXX serious), Mg_2_Si is crucial for strengthening the composite. Adding magnesium to aluminum reduces casting fluidity and lowers aluminum surface tension. In aluminum alloys, magnesium acts as a surfactant. In Al-Mg alloys, Mg tends to minimize the Al_2_O_3_ and form magnesium oxide (MgO).

$${\mathrm{Al}}_{2}O_{3}+3{\mathrm{Mg}}_{\left(1\right)}\to 3{\mathrm{MgO}}_{\left(s\right)}+2{\mathrm{Al}}_{\left(1\right)}$$

In short, the presence of Mg in AMMCs when the fabrication of composites separates the oxygen from the dispersoid surface enhances the dispersoid’s surface energy. However, its low ductility, poor fracture resistance, and highly reactive environment limit its use in the automobile industry. Minimal research has been carried out on AMMCs reinforced with MoS_2_ due to their poor wetting. MoS_2_, on the other hand, is chemically and thermally stable and has a high hardness and low density. It is also utilized to make bullet proof jackets, armor tanks, and other armored vehicles. Rao and Ramanaiah [5] investigated AA6061 AMMC reinforced with MoS_2_ and found that with the addition of MoS_2_, the composites’ hardness and tensile strength gradually increased. Senthil Kumar et al. [6] investigated the material’s porosity, micro-hardness, and compressive strength and found that increasing the MoS_2_ weight percentage increases micro-hardness and compressive strength.

Traditional machining processes have limitations such as machining an intricate shape, poor accuracy, energy consumption, recycling, etc. and nonconventional machining processes such as EDM, ECM, AWJM, and LBM are options which overcome the constraints [7,8]. WEDM can machine complex shapes, including hydraulic and injection mold parts, aeronautical structural parts, ejection dies, and shape tools [9]. In WEDM, machining is performed by thermoelectrical behavior between the workpiece and wire material (tool). Another advantage of WEDM is that there is no direct interaction between the tool and the work material; as a result, high-hardness materials can be machined conveniently, and only a simple clamping system is needed [10].

The performance of the WEDM depends on various parameters such as pulse off time, pulse on time, current, gap voltage, dielectric flow rate, wire feed, and tension. Srivastava et al. [11] explored the possibility of machining SiC reinforced Al2024 composites using the WEDM process. The experimental finding revealed that increases in peak current and pulse on-time increase the surface roughness while increases in reinforcement wt.% increase material removal rate (MRR).

Rani et al. [12] used the WEDM process to machine Al6061 reinforced with MoS_2_, and it is observed that the wire feed and pulse off time significantly contributed to the surface roughness, and pulse off-time and peak current influence the MRR. Saif and Tiwari [13] conducted machinability studies of AA6061 and AA5083 in WEDM machines. Surface roughness and MRR are explored and illustrated about pulse on time, pulse off time, and peak current. Surface roughness and MRR are highly influenced by pulse on time, and AA5083 has better MRR and surface roughness than AA6061. Similar machinability studies on aluminum metal matrix composites were reported and the output factors such as material removal rate, surface finish and overcut were investigated [14]. The overcut is one of the important factors for many applications, since it decides the geometrical accuracy of parts with intricate shapes and sizes. Due to the development of new materials recently, the machining process requires modifications and optimal settings. Therefore, the selection manufacturing process for any product is a significant challenge since each machining process has limitations and performance [15,16,17,18]. Selecting a suitable method among diverse machining processes is accomplished via multi-criteria decision-making (MCDM) and various optimization methods [19]. Dinesh Shinde et al. [20] analyzed MCDM models helps to the identification of the best alternative/courses of action in the presence of a group of evaluation criteria.

The machining/manufacturing process requires the best combination of different parameters to attain the desired product characteristics. As a result, this article began to look at MCDM techniques such as TOPSIS (order of preference by similarity to ideal solution), EDAS (Evaluation based on distance from average solution), and MOORA (multi-objective optimization based on ratio analysis) for determining the best setting for various machining parameters.

## 2. Materials and Methods

### 2.1. Fabrication of Composites

The matrix material in this study is pure aluminum (Al) powder with a particle size of 44 µm with a purity of 99.5 percent. On the other hand, magnesium (Mg) and molybdenum disulfide (MoS_2_) with a purity of 99 percent and particle sizes of 100 µm and 30 µm, respectively. The required aluminum/ 8% Mg and 3% MoS_2_ (AMM—Sample A), aluminum/6% Mg and 4% MoS_2_ (Sample B), aluminum/4% Mg and 5% MoS_2_ (Sample C), and composites are prepared by Powder Metallurgy process (PM). First, the ball milling and argon atmosphere are used to obtain the homogeneity and oxidation-free powder mixture; before ball milling, the required quantity of powder is measured in electronic balance with four-digit accuracy. Then, the powder mixture is heated in a furnace to ensure moisture freeness. Finally, a uniaxial hydraulic machine is used to make green compaction. The compaction pressure and sintering temperature are maintained at 650 MPa and 550 °C. The sintering is carried out under an argon atmosphere. The sintering time is 120 min, and the final part’s size is 18 mm and 27 mm in height.

### 2.2. Machining of Composites

**Figure 1:**
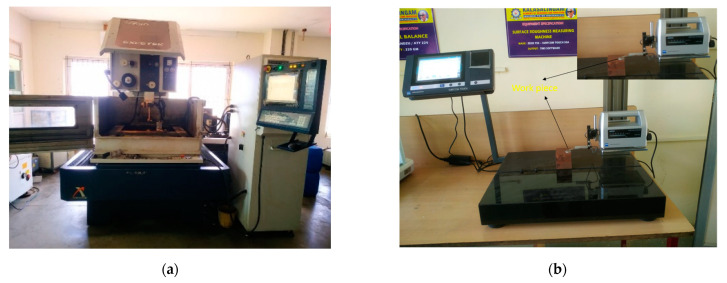
(**a**) Wire EDM experimental setup; (**b**) surface roughness measuring setup.

Figure 1a depicts the Wire-EDM machine (Model: Excetek V650, Excetek Technologies Co., Ltd, Taichung City, Taiwan) used to conduct the needed experiments. The cutting tool material is made with copper-zinc coated wire with a diameter of 0.25 mm. Wire feed rate of 3 mm/min is maintained constant for machining all the samples. As a high dielectric, deionized water is employed as a liquid medium. The three different samples (A, B, and C) are used to understand the machinability behavior of the composite materials. The experiment design is used to determine the correlation between the input and output parameters. The attributes level and ranges are listed in Table 1.

The input parameters were selected based on the priority in deciding the surface roughness, material removal rate, and overcut. Peak current and pulse on time are critical process parameters for controlling material removal rate, surface roughness, and polarity. The gap voltage is another important parameter for making the process stable and providing better surface roughness and material removal rate. The surface roughness of the composites is measured with Surfcom 50. The surface roughness is measured under the following conditions: 0.6 mm/s speed and a 5 mm travel distance. The surface roughness is measured in three locations, with the average value being used for analysis. The overcut of the sample is calculated by taking the hole’s photographic and importing it to measure the dimension in the CAD modeling packages. The various responses measured are shown in Table 2.

## 3. Optimization of Wire EDM Process

### 3.1. EDAS Technique

The evaluation based on distance from average solution (EDAS) technique was developed by Keshavarz Ghorabaee. In EDAS, positive and negative distances from the average solution evaluate alternatives. First, the average vector, the arithmetic average of the decision matrix, is calculated. Next, the average solution’s negative and positive distances are calculated by considering the type of criteria (benefit or cost). Next, weighted sums are calculated, and finally the normalization process is employed to obtain the final scores.

The calculation steps of EDAS can be summarized as follow.

Step 1: Create a decision matrix consisting of n criteria and m alternative as follow:

m—number of options
n—number of criteria
$x_{ij}$—value of criterion *j* at option *i*.

Step 2: Determine the average solution according to all criteria.

(1) $$AVG=\frac{\sum_{i=1}^{m}x_{i}}{m}$$

Step 3: Calculate the positive distance from average solution (PDA) and the negative distance from average solution (NDA) matrixes according to the type of criteria (benefit or cost) with Equations. If *j*th criterion is beneficial (benefit)

(2) $$PD_{ij}=\frac{max\;\left[0,\left(x_{ij}-AVG_{j}\right)\right]}{AVG_{j}}$$

if (j) is an indicator whose value is as high as possible

(3) $$ND_{ij}=\frac{max\;\left[0,\left(AVG_{j}-x_{ij}\right)\right]}{AVG_{j}}$$

if (j) is an indicator whose value is as high as possible and if *j*th criterion is non-beneficial (cost)

(4) $$PD_{ij}=\frac{max\;\left[0,\left(AVG_{j}-x_{ij}\right)\right]}{AVG_{j}}$$

if (j) is an indicator whose value is as low as possible

(5) $$ND_{ij}=\frac{max\;\left[0,\left(x_{ij}-AVG_{j}\right)\right]}{AVG_{j}}$$

if (j) is an indicator whose value is as low as possible

Step 4: Calculating the sum of positive distances (*SoP*) and the sum of negative distances (*SoN*).

(6) $$SoP_{i}=\sum_{i=1}^{m}w_{j}\;\cdot \;PD_{ij}$$

(7) $$SoN_{i}=\sum_{i=1}^{m}w_{j}\;\cdot \;ND_{ij}$$

$w_{j}$—weight of the criterion *j.*

Step 5: Normalizing the *SoP* and *SoN* values according to the formula.

(8) $$SSoP_{i}=\frac{SoP_{i}}{max\left(SoP_{i}\right)}$$

(9) $$SSoN_{i}=1-\frac{SoN_{i}}{max\left(SoN_{i}\right)}$$

Step 6: Calculating appraisement score (APS) of the options based on the formula.

(10) $$APS_{i}=\frac{1}{2}\left(SSoP_{i}-SSoN_{i}\right)$$

The rankings are calculated based on the above equations. The ranks of the respective samples have been shown in Table 3.

### 3.2. Optimization of Wire EDM Process Parameters

Table 4, Table 5, Table 6, Table 7, Table 8 and Table 9 shows the response and ANOVA table for samples A, B and C. In sample A, the contribution of the pulse on-time process parameter is slightly varying compared sample B and C whereas Samples B and C have similar contributions. But the appraisement score for samples A, B, and C is different. Similarly, the contribution percentage is also varied, but the order of contribution is similar for all the samples expect sample A (pulse on-time). From this, it could be understood that changing chemical composition does not alter the machining setting for the selected composite materials and their machining conditions—the optimal combination for all the samples set at I_3_, Ton_2_, and V_1_. To compare the efficiency of the proposed model, a confirmation experiment is carried out by randomly taking initial conditions. The randomly selected condition part of the experimental design is I_2_, Ton_3_, and V_3_.

### 3.3. Machining of Composites at Initial and Optimal Conditions

**Figure 2:**
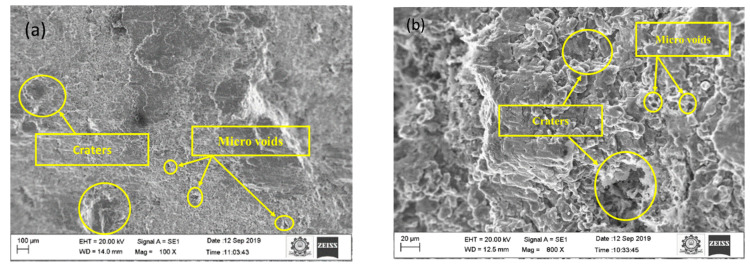
SEM image of machined surface at (**a**) 100X and (**b**) 800X at initial condition.

**Figure 3:**
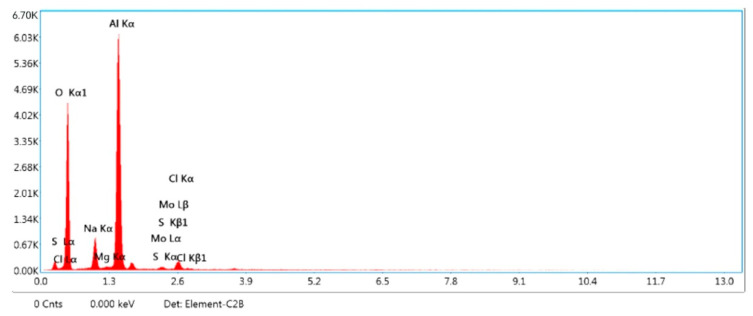
EDX image of machined surface initial conditions.

Figure 2a,b show the machined surface’s image at the initial condition for sample A. The following observation was made, the surface roughness and overcut of the composites are higher than the optimal setting for all the samples, according to Table 10. The optimal conditions provide a better surface finish and less overcut for all the samples. For sample A, the percentage of improvement is 6.67 % for surface roughness and 2.07 for overcut. For sample B, the percentage of improvement is 2.25 % for surface roughness and 1.92 for overcut. For sample C, the percentage of improvement is 2.67% for surface roughness and 0.95 for overcut. Figure 3 shows the EDX image of the machined surface at the initial condition, though there are effects in surface roughness and overcut. Still, no significant reaction is observed at a higher temperature within the metal and electrode.

**Figure 4:**
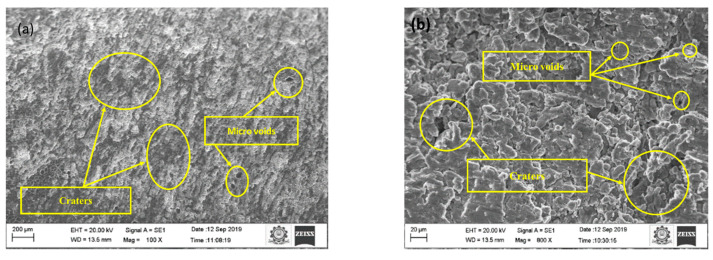
SEM image of machined surface at (**a**) 100X and (**b**) 800X at optimal conditions.

Sinkholes, agglomerates, micro-voids, and micro-fissures affect the machined surface. Initial conditions and optimal conditions analyze machined surfaces. Under the initial and optimal conditions, Sample A is represented in Figure 2a,b and Figure 4a,b. When comparing the initial and ideal parameters, the size of the sinkholes and agglomerates is noteworthy. Since the selected range of pulse on time is large, the discharge energy is considerable at first, as a result, the MRR is high. Higher discharge energy for a longer duration is provided by increasing pulse on time and peak current. Thermoelectric behavior removes more material with increased discharge energy and duration [21,22,23,24,25]. The removed debris particle would stick between the workpiece and electrode, which causes the additional load to the already machined surface.

The thermal load and load due to the debris causes more considerable stress than the ultimate stress of sample A, leading to micro cracks. The experiment results also show that MoS_2_ increases the surface roughness irrespective of the parameter selected. This could be that during machining, MoS_2_ is not melted, and the un-melted particles become stuck on the workpiece surface, lowering the surface quality. 

The same is indicated as the reason for an increase in the overcut. A discrete electric pulse amplifies a severe intensification in peak current and pulse on time, resulting in the formation of a degraded surface [26,27,28]. The secondary sparking energy frequently occurs during machining at this moment, and as a result, the overcut is increased. When the debris is not removed effectively by the dielectric medium, it can solidify on the workpiece surface, resulting in an uneven surface. At a high peak and pulse on time, it causes micro-cracks and voids [29]. At the initial condition, the number of micro-voids and craters were observed in Figure 2a,b. The other reason could be that more metal erosion and melting occurs in the machining zone due to the high production of spark energy due to electrostatic and electromagnetic forces at maximum peak current. Therefore, the surface deteriorates. In ideal circumstances, the number of sinkholes and micro-voids is lower than at the initial condition. The established plasma channel is reinforced by an extended pulse on time [30,31,32]. The gap voltage increases, the surface deteriorates with pits, and sinkholes and voids grow in size. As the gap voltage rises, the discharge energy between the spark gaps increases. The surface area is increased by removing excess materials from the workpiece [33,34,35,36,37].

**Figure 5:**
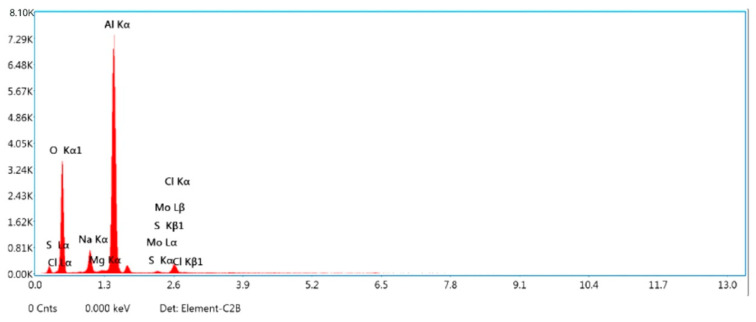
EDX image of machined surface under optimal conditions.

Furthermore, the discharge cycle is reduced at greater gap voltages, resulting in a low-quality surface. Figure 5 displays the EDX image of the machined surface in its initial state, with surface roughness and overcut effects. However, no appreciable reaction is recorded at higher temperatures within the metal and electrode.

**Figure 6:**
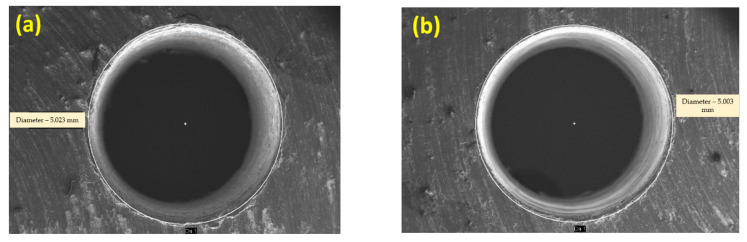
(**a**) SEM image of overcut initial conditions; (**b**) optimal conditions.

Figure 6 shows the SEM image of shape of the machined hole at initial and optimal conditions for sample A. It is found that at optimal conditions the size of the over cut deviation is less compared to the initial conditions (since, in the randomly selected conditions, the gap voltage is fixed at higher level). At higher level over cut values amplifies the size of the required hole. The amount of energy generated for the selected gap voltage discharges is at higher energy level along with pulse on-time and peak current. This leads to quick melting of the surface which in turn produces larger discharge gap and deep crater at the surface of the entry and exit region. The size of the over cut is increasing as reinforcement content increases, as is evident from the experimental results of other samples.

## 4. Conclusions

The following findings were made while machining AMMC with a Wire EDM machine.

- The addition of MoS_2_ decreases the surface roughness and overcut of the composite’s materials due to the melting point difference and sticking property of MoS_2_ at higher working temperatures.
- EDAS is optimizing process parameters to obtain better surface roughness and overcut. This technique is applied to three samples fabricated by varying percentages of MoS_2_. It is observed that the steps for all the processes, including weightage used for output performance, are kept constant.
- The optimal process parameters are set at I_3_, Ton_2_, V_1_. Therefore, the increase in MoS_2_ content increases the machining performance of the composites and does not alter the optimal parameter setting range.
- Among the different process parameters, the contribution of pulse on-time and peak current plays a vital role in affecting the machining process.

## Figures and Tables

**Table 1:** WEDM parameters and their levels.

Control Factor	Unit	Symbol	Range	Level 1	Level 2	Level 3
Peak Current	A	I	8–12	8	10	12
Pulse on-time	µs	$T_{on}$	10–20	10	15	20
Gap voltage	V	V	10–30	10	20	30

**Table 2:** Experimental results for samples A, B, and C.

I	T_on_	V	R_a_ µm	OC mm	R_a_ µm	OC mm	R_a_ µm	OC mm
			Sample A		Sample B		Sample C	
1	1	1	7.4320	0.4200	6.3713	0.4957	6.9463	0.3614
1	2	2	7.5672	0.5301	6.5439	0.5827	6.9626	0.4449
1	3	3	7.5339	0.4623	6.6167	0.6723	6.9752	0.5425
2	1	2	7.5974	0.6957	6.7492	0.7752	6.9693	0.6461
2	2	3	7.5624	0.7356	6.8493	0.8513	6.9193	0.7223
2	3	1	7.5165	0.7756	6.9142	0.8770	6.9956	0.7449
3	1	3	7.5794	0.8356	6.9317	0.9270	6.9379	0.7991
3	2	1	7.5654	0.8926	7.4371	0.9578	6.9817	0.8313
3	3	2	7.6158	0.5213	7.9955	0.4823	6.9765	0.3489

**Table 3:** EDAS Ranking.

S. No	Sample A		Sample B		Sample C	
	EDAS	Rank	EDAS	Rank	EDAS	Rank
1.	0.5000	2	0.4624	6	0.4765	3
2.	0.2542	7	0.3023	12	0.3108	6
3.	0.3946	3	0.1625	15	0.1230	8
4.	0.0983	9	0.1331	16	0.0926	9
5.	0.1746	8	0.2806	13	0.2679	7
6.	0.2619	6	0.3229	9	0.3150	5
7.	0.3846	4	0.4313	8	0.4337	4
8.	0.5010	1	0.5729	1	0.5032	1
9.	0.2811	5	0.5000	3	0.5000	2

**Table 4:** Response table for sample A.

	I	Ton	V
Level 1	0.3829	0.3276	0.4206
Level 2	0.1783	0.3096	0.2112
Level 3	0.3886	0.3125	0.3179
Delta	0.2103	0.0180	0.2094
Rank	1	2	3

**Table 5:** Analysis of variance for sample A.

	DF	Adj SS	Adj MS	Contribution In %
I	2	0.086146	0.043073	55.96
Ton	2	0.056221	0.000281	39.19
V	2	0.065802	0.032901	42.74
Error	2	0.001434	0.000717	0.93
Total	8	0.153944		100

**Table 6:** Response table for sample B.

	I	Ton	V
Level 1	0.3091	0.3423	0.4527
Level 2	0.2455	0.3853	0.3118
Level 3	0.5014	0.3285	0.2915
Delta	0.2559	0.0568	0.1613
Rank	1	3	2

**Table 7:** Analysis of variance for sample B.

	DF	Adj SS	Adj MS	Contribution In %
I	2	0.106496	0.053248	58.70
Ton	2	0.046283	0.002633	25.51
V	2	0.005266	0.023141	12.89
Error	2	0.023375	0.011688	2.900
Total	8	0.181420		100.00

**Table 8:** Response table for sample C.

	I	Ton	V
Level 1	0.3034	0.3343	0.4316
Level 2	0.2252	0.3606	0.3011
Level 3	0.4790	0.3127	0.2749
Delta	0.2538	0.0480	0.1567
Rank	1	2	3

**Table 9:** Analysis of variance for sample C.

	DF	Adj SS	Adj MS	Contribution In %
I	2	0.101352	0.050676	52.12
Ton	2	0.047391	0.001731	24.37
V	2	0.042258	0.021129	21.73
Error	2	0.003463	0.023696	1.780
Total	8	0.194463		100

**Table 10:** Percentage improvement in performance at optimal condition.

Description	Input Parameters	Ra	OC	Ra	OC	Ra	OC
		Sample A		Sample B		Sample C	
Initial Setting	I_2_, Ton_3,_ V_3_	7.9622	0.9781	7.1425	0.8476	7.7731	0.9012
Optimal Setting	I_3_, Ton_2,_ V_1_	7.4371	0.9578	6.9817	0.8313	7.5654	0.8926
% Of improvement		6.67%	2.07%	2.25%	1.92%	2.67%	0.95%

## Data Availability

Not applicable.
